# Competition and disturbance affect elevational distribution of two congeneric conifers

**DOI:** 10.1002/ece3.8647

**Published:** 2022-02-19

**Authors:** Koichi Takahashi, Keigo Ikeda, Isao Okuhara, Rintaro Kurasawa, Suguru Kobayashi

**Affiliations:** ^1^ Department of Biology Faculty of Science Shinshu University Matsumoto Japan; ^2^ Institute of Mountain Science Shinshu University Matsumoto Japan; ^3^ Graduate School of Science and Technology Shinshu University Matsumoto Japan

**Keywords:** *Abies*, growth, long‐term observation, mortality, range limit, recruitment, regeneration dynamics, tree‐ring width

## Abstract

Climatic change will affect elevational vegetation distribution because vegetation distribution is related to thermal conditions. However, how elevational species distributions are determined by biotic and abiotic factors is not clear. The long‐term plot census along an elevational gradient is indispensable to clarify mechanisms of elevational distribution of tree species. Two congeneric conifers, the less shade‐tolerant *Abies veitchii* and shade‐tolerant *A*. *mariesii*, dominate at low and high elevations, respectively, in the subalpine zone in Japan. This study investigated the population dynamics of the two species at three elevations (low, middle, high) for 13 years to examine why the two species dominated the different elevations from the viewpoints of competition and disturbance. This study showed that growth and survival rates were not highest at the most dominant elevations for each species. At the high elevation where *A*. *mariesii* dominated and small disturbances frequently occurred, the recruitment rate of *A*. *mariesii* was highest among the three elevations and that of *A*. *veitchii* was largely decreased by tree competition. However, *A*. *veitchii* was dominant earlier than *A*. *mariesii* at the low elevation after large disturbances by the high growth rate of individual trees. Therefore, *A*. *mariesii* was superior to *A*. *veitchii* at the high elevation because of its high recruitment rate and large reduction of recruitment of *A*. *veitchii* due to competition, while *A*. *veitchii* was superior to *A*. *mariesii* at the low elevation after large disturbances because of higher growth rate than *A*. *mariesii*. It is suggested that the elevational distributions of the two species were determined by elevational changes in population dynamics in relation to competition and disturbance. Long‐term observational studies of forest dynamics among various elevations are indispensable to predict the effects of climatic change on vegetation distribution.

## Graphical abstract

Climatic change will affect elevational vegetation distribution because vegetation distribution relates to thermal conditions. Two congeneric conifers, less shade‐tolerant *Abies veitchii* and shade‐tolerant *A*. *mariesii* dominate at low and high elevations, respectively, in the subalpine zone, Japan. This study showed that the elevational distributions of the two species were determined by elevational changes in regeneration traits in relation to competition and disturbance.

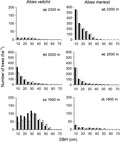

## 1 INTRODUCTION

The elevational change of plant distributions is one of the main drivers for species diversity. The abundant‐center hypothesis, explaining species distribution along elevational and latitudinal gradients, predicts that each species is replaced by other species at the distribution limits because the performance of each species is highest at the center of the geographical distribution and decreases toward both distribution limits due to unfavorable abiotic conditions (Angert & Schemske, 2005; Brown, 1984; Guo et al., 2005; Hengeveld & Haeck, 1982). Some researchers describe the asymmetric abiotic stress limitation hypothesis that upper and lower distribution limits are determined by abiotic and biotic factors, respectively (Cahill et al., 2014; Connell, 1961; Koehler et al., 2012; MacArthur, 1972; Molina‐Montenegro et al., 2012; Woodward, 1986). This hypothesis assumes the trade‐off relationship between growth and cold tolerance (Battisti et al., 2005; Hersteinsson & Macdonald, 1992; Koehler et al., 2012; Molina‐Montenegro et al., 2012; Wethey, 2002). The acquisition of cold tolerance is thought to need a cost of resource, which results in lower growth rates (Koehler et al., 2012). According to the two hypotheses, the performance of a species at the distribution limits is lower than that at the center of the distribution or than that of the sympatric species at the center of the distribution, which causes the competitive exclusion of the population and forms the distribution limit. Therefore, species cannot maintain the population beyond the distribution limits, and the elevational distribution is stably maintained. However, few studies have explicitly examined elevational change of population dynamics of tree species in order to clarify the change of distribution with elevation. Such information is also important for studies of changes of species distribution due to climate change because many studies simply assume that species distribution shifts with climatic conditions (Iverson & Prasad, 1998; Thuiller et al., 2005).

Population dynamics of a species at a given site is determined by three demographic rates (growth, recruitment, and survival). The three demographic rates are affected not only by elevation but also by competition. Sessile plants compete for resources only with neighboring individuals (Castagneri et al., 2010; Getzin et al., 2006; Takahashi, 1996, 2010a). Therefore, local crowding of neighboring trees (i.e., competition) affects the three demographic rates, which further influences the population dynamics along elevational gradients. The annual growth rate of individual trees of a certain species often decreases with elevation because of short growth periods due to low temperature (Coomes & Allen, 2007; Takahashi, 2003, 2021). However, information of elevational changes in survival and recruitment rates is limited, compared with the growth rate (Takahashi, 2010a). The abundant‐center hypothesis predicts that three demographic rates (growth, survival, and recruitment) of a species are greater at the most dominant elevation. It is also expected that the three demographic rates of a species are largely decreased by local crowding (i.e., competition) at physiologically unfavorable elevations (i.e., distribution edges) because of the double stresses of competition at unfavorable elevations.

On the other hand, it is possible that the current demographic rates (growth, survival, and recruitment) of a species are not always highest at the most dominant elevation. Disturbance is thought to be a factor affecting demographic rates. Large disturbances, such as typhoons and forest fires, sometimes occur in forest ecosystems (Pickett, 1980). Furthermore, there is a trade‐off between survival in low‐light conditions (i.e., shade tolerance) and growth in full‐light conditions (Kunstler et al., 2009; Poorter & Arets, 2003). Thus, a large disturbance greatly influences forest regeneration and species composition by reducing stand biomass and tree density (i.e., competitive interactions, Firm et al., 2009; Loehle, 2000; Papaik & Canham, 2006; Poulson & Platt, 1996; Takahashi et al., 2006; Takahashi et al., 2007).

Stand development after a large disturbance is faster in more productive sites (Larson et al., 2008). Productivity is greater in lower elevations (Takahashi, 2021). The rapid dominance of shade‐intolerant fast‐growing species after large disturbances is probably conspicuous at lower elevations because the long growth period at low elevations increases the interspecific difference in annual growth rates between shade‐intolerant fast‐growing species and shade‐tolerant slow‐growing species. Leaf longevity of shade‐intolerant species is often shorter than that of shade‐tolerant species and needs more annual carbon gain to maintain whole‐plant leaf mass (King, 1994; Takahashi & Obata, 2014). The short growth period at high elevations is unfavorable for annual production of shade‐intolerant species with short leaf longevity, i.e., shade‐intolerant species are less adapted to high elevations than shade‐tolerant species if interspecific differences in the other physiological and ecological traits are small. Therefore, it is expected that both disturbance and shade tolerance influence the elevational vegetation distribution through species differences in three demographic rates of growth, survival, and recruitment (Pollmann & Veblen, 2004).

Two evergreen conifers, *Abies veitchii* Lindl. and *A*. *mariesii* Mast., dominate in subalpine forests in central Japan (Franklin et al., 1979). The two *Abies* species are shade tolerant, but *A*. *veitchii* is less shade tolerant and dominates after large disturbances because of faster growth rate than *A*. *mariesii* (Kohyama, 1983, 1984; Takahashi & Obata, 2014). *A*. *veitchii* and *A*. *mariesii* dominate low and high elevations of the subalpine zone, respectively, and codominate the mid‐elevation (Aizawa & Kaji, 2003). However, the reason that the two *Abies* species dominate different elevations is unknown. Therefore, this study examined how the elevational distributions of the two *Abies* species are formed by using the 13‐year observation data of permanent plots at three elevations. Specifically, we addressed the following two questions:

1. Are the three demographic rates (growth, survival, and recruitment) of *A*. *veitchii* and *A*. *mariesii* highest at low and high elevations, respectively, and/or are largely decreased by local crowding (i.e., competition) at unfavorable high and low elevations, respectively?
2. Does *A*. *veitchii* dominate low elevations after a large disturbance by higher growth rate than *A*. *mariesii*?

## 2 MATERIALS AND METHODS

### 2.1 Study sites

This study was undertaken at 1600 m, 2000 m, and 2300 m above sea level on the east‐facing slope of Mt. Norikura (36˚06'N, 137˚33'E, 3026 m a.s.l.) in central Japan. The study sites were located in Chubu Sangaku National Park, established in 1934. There was no stump at the study sites, indicating no evidence of logging. The elevation 1600 m a.s.l. is the lowest range limit of the subalpine zone. The timberline is located at 2500 m a.s.l. (Takahashi et al., 2012). Meteorological data of a decade (2008‒2017) of the study sites were obtained from the Agro‐Meteorological Grid Square Data, NARO (Kominami et al., 2019; Ohno et al., 2016) (https://amu.rd.naro.go.jp/) (1 × 1 km resolution). Mean annual temperatures were 4.8, 3.6, and 0.6°C at 1600 m, 2000 m, and 2300 m a.s.l., respectively. Mean annual precipitation was slightly greater at higher elevations, i.e., 2548, 2596, and 2700 mm year^−1^ at 1600 m, 2000 m, and 2300 m a.s.l., respectively. The maximum snow depth was also greater at higher elevations, i.e., 128, 140, and 180 cm at 1600 m, 2000 m, and 2300 m a.s.l., respectively. Annual solar radiation was slightly lower at higher elevation, i.e., 733, 719, and 684 MJ m^−2^ year^−1^ at 1600 m, 2000 m, and 2300 m a.s.l., respectively.

The study sites were dominated by two conifers, *Abies mariesii* and *A*. *veitchii*. Although *Tsuga diversifolia* Mast. and *Picea jezoensis* var. *hondoensis* Rehder also grow at the study sites, the abundances were lower than that of the two *Abies* species (Miyajima et al., 2007). Subordinate trees were all deciduous broad‐leaved trees such as *Betula ermanii* Cham. and *Sorbus commixta* Hedland. Although the two *Abies* species are distributed between 1600 m and 2500 m a.s.l. (Miyajima & Takahashi, 2007), *A*. *veitchii* and *A*. *mariesii* dominate between 1600 m and 2200 m a.s.l. and between 2000 m and 2500 m a.s.l., respectively (Miyajima et al., 2007).

### 2.2 Plot survey

The three demographic rates (growth, survival, and recruitment) of tree species are obtained from the permanent plot census (Kohyama, 1993; Luo & Chen, 2011; Lutz et al., 2014; Pacala et al., 1996). One plot of 100 m × 100 m was established at 1600 m and another 2300 m a.s.l. in 2004, and a plot of 50 m × 120 m was established at 2000 m a.s.l. in 2006. Each plot was divided into contiguous 10 × 10 m quadrats. All trees ≥5.0 cm diameter at breast height (DBH) were tagged. The species was identified, and the coordinate within a quadrat was measured. DBH was measured at the three plots when the plots were established and again in 2008, 2011, and 2016. Dead trees were recorded during two successive censuses and were classified as standing dead, stem broken, and uprooted trees. Standing dead trees were assumed to have died of suppression or senescence, and dead trees that included stem broken and uprooted trees were assumed to have died of disturbances (Nakashizuka et al., 1992; Takahashi, 2010a). Although stem broken and uprooted trees often occur after being standing dead trees, this classification (standing dead trees or not) is a convenient measure of suppression, senescence, or disturbance for tree mortality. New recruitment trees growing to ≥5.0 cm DBH during two successive censuses were also recorded.

### 2.3 Wood core sampling

To investigate the occurrence of large disturbances and growth releases following the disturbances, wood cores (5 mm in diameter) were sampled at breast height (1.3 m) from thirty‐five large canopy trees of *A*. *veitchii* at 1600 m a.s.l., *A*. *veitchii* and *A*. *mariesii* at 2000 m a.s.l. in 2006, and *A*. *mariesii* at 2300 m a.s.l. in 2003. DBH of cored trees was between 27 and 61 cm at the three elevations.

Increment cores were cut transversely into 1.5 mm thick strips by using a twin‐bladed saw. The strips were oven‐dried and then they underwent soft X‐ray analysis at 20 kV and 14 mA for 210 s from a distance of 2.2 m; a calibration wedge was also used in the soft X‐ray analysis. The resultant radiographs were scanned at resolution 2400 dpi. The tree‐ring widths were measured by using a WinDENDRO (Regent Instruments Inc., Quebec City, Canada).

All cores were cross‐dated visually by matching characteristic wide and narrow rings that were synchronous within sample trees. Visual cross‐dating was statistically verified by using the COFECHA program (Holmes, 1983, 1994) that tests each individual series against a master dating series (mean of all series) from correlation coefficients.

## 3 DATA ANALYSIS

### 3.1 Effects of elevation and competition on three demographic rates

To examine question 1, the effects of elevation and competition on the three demographic rates (growth, survival, and recruitment rates) of the two *Abies* species were analyzed. Competition was expressed as local crowding of neighboring trees for each target tree that the growth and survival were analyzed. This study defined the neighborhood area as a 10 × 10 m quadrat in which the target tree was located (Kohyama, 1992, 1993; Takahashi & Kohyama, 1999). All trees greater than 5 cm DBH within a 10 × 10 m quadrat were treated as neighboring trees. Local crowding was calculated as a sum of the basal area of all neighboring trees for each target tree. However, this definition of neighborhood area sometimes brings about a crude measure of local crowding if target trees are near the edge of a quadrat, i.e., a target tree near the edge of a quadrat will be affected by trees at the adjacent quadrat but not much by trees at the other end of the same quadrat. The degree of competition ability of neighboring trees to the growth of target trees also decreases with increase of the distance from the target tree. In the preliminary analysis, this study compared the fitness of regression models for the growth of target trees between models with and without the distance from target trees. However, the fitness of regression models was almost the same between the models with and without the distance (Appendix S1). Furthermore, trees near the plot edge cannot be used as target trees for the model with the distance because local crowding cannot be calculated (i.e., the number of trees that can be used for the analysis decreases). The problem of the decrease of the number of trees is more pronounced in the analysis of mortality and recruitment rates than growth because of the small number of dead and recruitment trees. Therefore, this study defined the neighborhood area as 10 × 10 quadrat in which the target tree was located.

Certain species have inhibitory effects such as allelopathy on the growth of other species (Gómez‐Aparicio & Canham, 2008; Newman & Rovira, 1975; Yuan et al., 2012). In this case, the effect of local crowding on the growth of target plants is different between neighboring species. However, the effect of species identity was not detected among dominant species in our preliminary analysis (Appendix S2). Random spatial distributions among the dominant species probably caused the weak effect of species identity on the growth of target trees (Appendix S3). Therefore, we calculated local crowding of neighboring trees without consideration of species identity.

Data were divided into two periods, early period (2004–2011 [or 2006–2011 at 2000 m a.s.l.]) and latter period (2011–2016). Data of the two periods were used to calculate the following Equations (1), (2), (3). Absolute diameter growth rate (ADGR, cm year^−1^) was calculated for each surviving tree during early and latter periods by DBH growth rate (cm) divided by the observation period (years). We examined the effects of elevation and local crowding on ADGR by the generalized linear mixed model. ADGR is often size‐dependent (Takahashi, 2010a). Furthermore, the response of ADGR to elevation is possibly different depending on the tree size and local crowding, so interactions between tree size (DBH), elevation, and local crowding were included as independent variables of the model. Therefore, the model of ADGR*_i_* of species *i* is as follows:

(1) $$\begin{array}{cc} {\text{ADGR}}_{i} & =a_{0}+a_{1j}+a_{2}\ln\text{DBH}+a_{3}\sum \text{BA}+a_{4}\ln\text{DBH}\times \sum \text{BA}\\ & +a_{5j}\ln\text{DBH}+a_{6j}\sum \text{BA} \end{array}$$

where *a*_0_–*a*_6_ *_j_* are coefficients, ΣBA (cm^2^ m^−2^) is the total basal area (cm^2^) of neighboring trees within a quadrat divided by the quadrat area (100 m^2^), and ln is the natural logarithm. Coefficients *a*_1_ *_j_*, *a*_5_ *_j_*, and *a*_6_ *_j_* are categorical variables of elevation *j*, and coefficient *a*_1_ of 1600 m a.s.l. is zero. Individual trees were treated as random effects.

Mortality was analyzed by the generalized linear model with logistic regression because death is a discrete event (0: survival, 1: death). Local crowding of neighboring trees (ΣBA) and tree size (DBH) were included as independent variables. Size‐dependent mortality sometimes shows a U‐shaped pattern, with higher mortality of small trees and large canopy trees because of suppression of small trees and because of senescence and killing by disturbances of large trees (Monserud & Sterba, 1999; Takahashi & Kohyama, 1999). Such a U‐shaped mortality can be expressed by a quadric function of size (Takahashi, 2010a). Combining these effects, the hypothesized mortality model is:

(2) $$M_{i}=\frac{100}{1+\exp\left(‐\left(b_{0}+b_{1j}+b_{2}\text{DBH}+b_{3}{\text{DBH}}^{2}+b_{4}\sum \text{BA}+b_{5}\ln\left(\text{yr}\right)\right)\right)}$$

where *M_i_* is a percent mortality per observation period (yr, years) for species *i*, and *b*_0_–*b*_5_ are coefficients for independent variables. Coefficient *b*_1_ *_j_* is a categorical variable of elevation *j*, and coefficient *b*_1_ of 1600 m a.s.l. is zero. ΣBA (cm^2^ m^−2^) is the total basal area (cm^2^) of neighboring trees within a quadrat divided by the quadrat area (100 m^2^).

The number of recruitment trees into ≥DBH 5 cm during two successive censuses was counted for each 10 × 10 m quadrat. It is expected that the recruitment rate is proportional to the amount of parent trees (Uriarte et al., 2005) and that larger trees produce more seeds (Kohyama, 1982; Seki et al., 2013). Seeds possibly come from the outside of the 10 × 10 m quadrat. Furthermore, there is a time lag between seed dispersal and the recruitment into ≥5 cm DBH. Therefore, the number of recruitment trees within each 10 × 10 m quadrat was assumed to be proportional to the total basal area (cm^2^ m^−2^) of the species within a whole plot (i.e., 1 ha for 1600 m and 2300 m a.s.l., 0.6 ha for 2000 m a.sl.). The recruitment rate was analyzed by the following equation:

(3) $$R_{i}=\sum {\text{BA}}_{i}\;\exp\left(c_{0}+c_{1j}+c_{2}\sum \text{BA}+c_{3}\text{yr}+c_{4j}\sum \text{BA}+1\right)$$

where *c*_0_–*c*_4_ *_j_* are coefficients of independent variables. *R_i_* is the recruitment rate (trees ha^−1^) of species *i* per observation period (yr, years), ΣBA (cm^2^ m^−2^) is the total basal area (cm^2^) of neighboring trees within a quadrat divided by the quadrat area (100 m^2^). ΣBA*_i_* (cm^2^ m^−2^) is the total basal area of species *i* in the whole plot divided by the plot area. Coefficient *c*_1_ *_j_* and *c*_4_ *_j_* are categorical variables of elevation *j*, and these coefficients are zero for 1600 m a.s.l. The value of *R_i_* divided by ΣBA*_i_* is the *per*‐*capita* recruitment rate (Kohyama, 1992). This study compared the recruitment rate per species basal area among three elevations for each species.

Selection of independent variables was performed by Akaike information criteria (AIC) for (1), (2), (3), and the model with the lowest AIC was selected as being the best for each equation. A model with the lowest value of AIC is essentially the best model. However, any model is substantially the same as the best model with the lowest value of AIC if the difference in AIC value (ΔAIC) of the model of interest is smaller than 2 as compared with the best model (Burnham & Anderson, 2002). In general, the growth and recruitment rates are decreased, and the mortality is increased by local crowding (Castagneri et al., 2010; Kohyama, 1992, 1993; Luo & Chen, 2011). The number of dead trees and recruitment trees is expected to be proportional to the observation period. Therefore, the model with the lowest AIC among the models with ΔAIC within 2 and that selected local crowding was used for the growth model (Equation 1), and the model with the lowest AIC among the models with ΔAIC within 2 and that selected both local crowding and observation period was used for the mortality and recruitment models (Equation (2), (3)).

### 3.2 Observed mortality and recruitment rate

To examine question 2, the population dynamics of *A*. *veitchii* and *A*. *mariesii* were examined in relation to disturbances at each elevation. Mortality (% year^−1^) was calculated for the early period (2004–2011 [or 2006–2011 at 2000 m a.s.l.]) and the latter period (2011–2016) by the following equation:

(4) $$\text{Mortality}=\left\{\ln\left(N_{i}/N_{s}\right)\right\}\times 100/\text{yr}$$

where *N_i_* is the initial number of trees of each measurement period, and *N_s_* is the number of surviving trees by the end of the measurement period. The observation period (yr) was 7 years (or 5 years at 2000 m a.s.l.) for the early period and 5 years for the latter period. A significant difference in mortality between the two periods was tested by 95% confidence intervals generated using a 1000 iteration bootstrap technique (Crowley, 1992). Furthermore, trees were divided into five DBH classes (5.0–9.9, 10.0–19.9, 20.0–29.9, 30.0–39.9, 40.0 ≤ DBH). Mortality was compared between mortality forms (standing dead trees, stem broken, and uprooted trees) for each size class by using the pooled data of all species.

Recruitment rate (% year^−1^) was calculated for each of the early period (~2011) and the latter period (~2016) for each species by the following equation:

(5) $$\text{Recruitment rate}=\left\{\ln\left(N_{f}/N_{s}\right)\right\}\times 100/\text{yr}$$

where *N_f_* is the sum of the initial number of trees at the beginning of the measurement period and new recruitment trees, *N_s_* is the number of surviving trees during the measurement period, and yr is the observation period (7 years [or 5 years at 2000 m a.s.l.] for the early period, and 5 years for the latter period). A significant difference in recruitment rates between the two periods was tested by 95% confidence intervals generated using a 1000 iteration bootstrap technique (Crowley, 1992).

### 3.3 Detection of release events of tree growth

Release events of growth were detected by using the R package TRADER (Altman et al., 2014) that used the method of Nowacki and Abrams (1997) for tree‐ring width chronologies of two *Abies* species at the three elevations. In this method, the average tree‐ring width over the immediately preceding 10 years, *M*_1_ (including the target year), and the average tree‐ring width over the immediately following 10 years, *M*_2_ (excluding the target year), are computed, and the percentage growth change is obtained as {(*M*_2_–*M*_1_)/*M*_1_} ×100. The minimum thresholds applied for releases are typically a 25% and a 50% growth change for moderate and major releases, respectively (Altman et al., 2014). We counted the number of trees with growth change ≥25% (i.e., moderate plus major releases) of each year for each species at each elevation. The results of growth releases were summarized in 5‐year intervals, which minimized the bias caused by measurement errors and the lag of tree response to disturbances (Altman et al., 2014; Lorimer & Frelich, 1989). All statistical analyses were performed by free software R ver. 3.3.3 (R Core Team, 2017).

## 4 RESULTS

### 4.1 Effects of elevation and competition on three demographic rates

**FIGURE 1:**
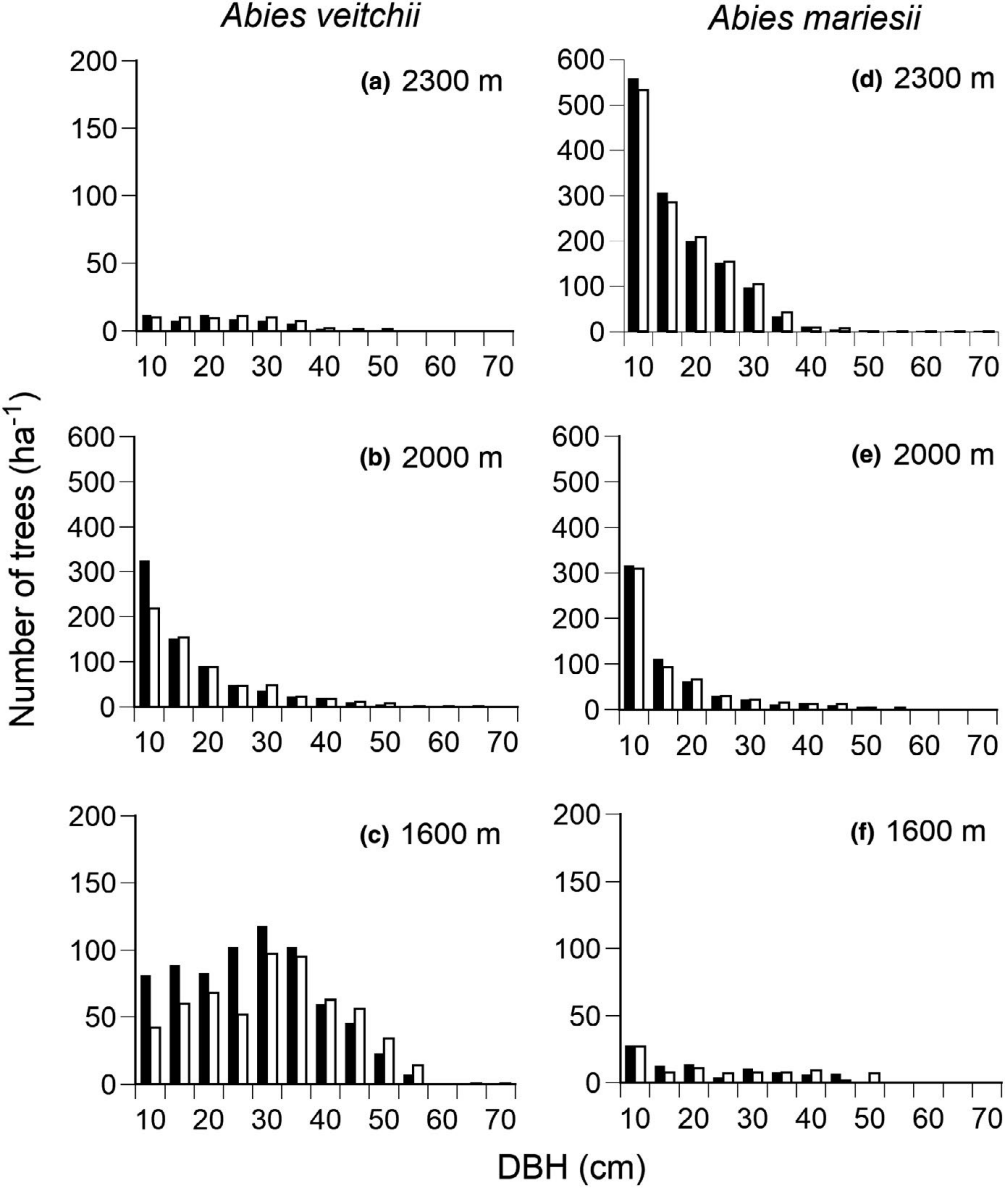
Frequency distributions of diameter at breast height (DBH) of *Abies veitchii* (a–c) and *A*. *mariesii* (d–f) at three elevations (1600 m, 2000 m, and 2300 m a.s.l.). Solid and open bars indicate the first census (2004 at 1600 m and 2300 m a.s.l., 2006 at 2000 m a.s.l.) and the final census (2016), respectively. Note the scale of the vertical axis is different in *Abies veitchii* at 2000 m a.s.l. and *A*. *mariesii* at 2000 m and 2300 m a.s.l. from that in the other Figures (the maximum value is 200)

**FIGURE 2:**
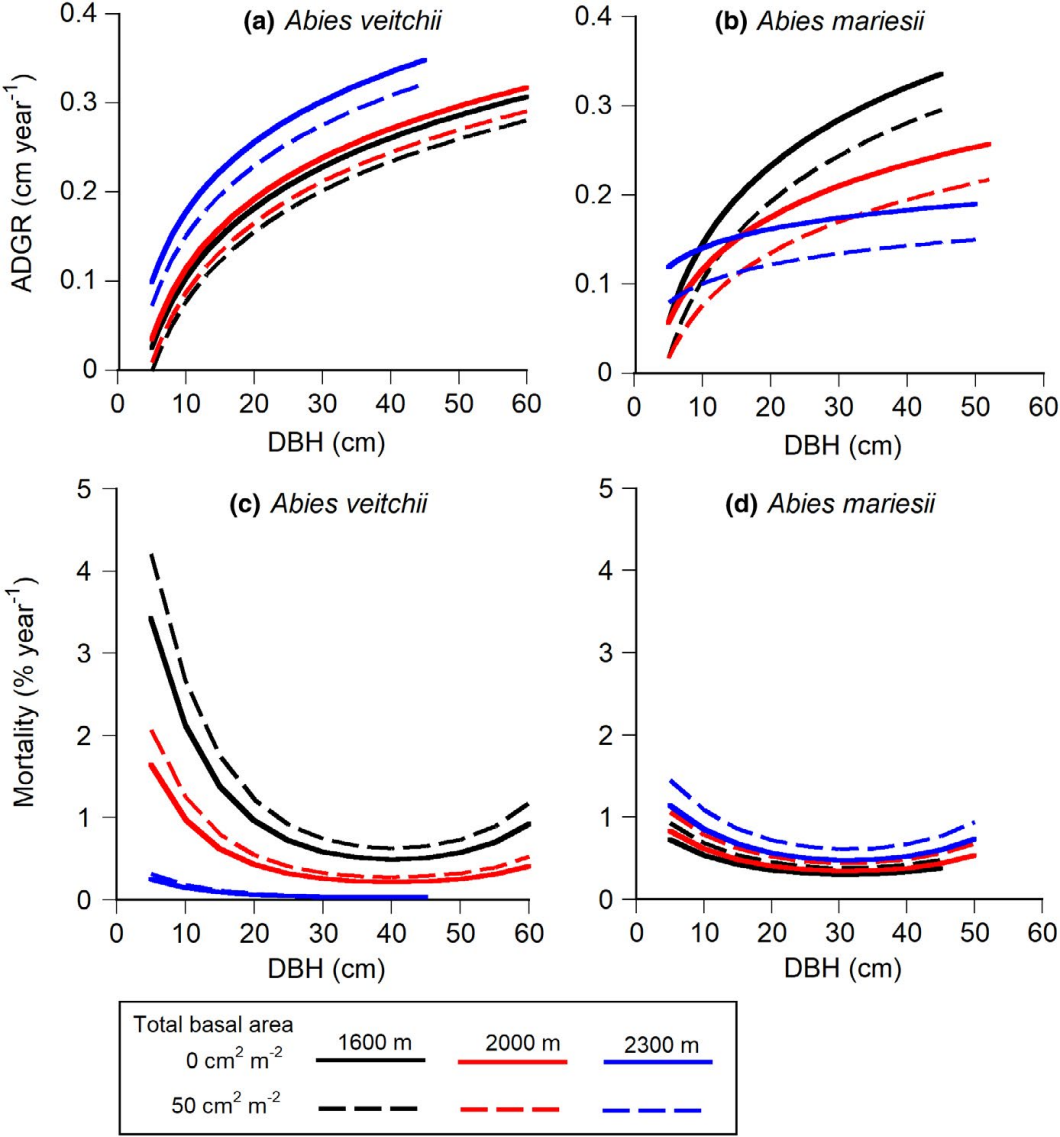
Estimated absolute diameter growth rates (ADGR) (a, b) and mortalities (c, d) of *Abies veitchii* and *A*. *mariesii* to DBH in two local crowding conditions (solid and broken lines indicate 0 and 50 cm^2^ m^−2^ of total basal area of neighboring trees, respectively) for each elevation. Black, red and blue lines indicate 1600 m, 2000 m, and 2300 m a.s.l., respectively. Regression lines of each species were drawn up to the observed maximum DBH at each elevation. See Appendix S4: Table S4.5 for equation parameters

Abundance of *A*. *veitchii* decreased from 1600 m to 2300 m a.s.l., while that of *A*. *mariesii* increased (Figure 1, Appendix S4: Table S4.4). We investigated the effects of elevation and competition on growth, mortality, and recruitment rates to examine question 1, i.e., whether the three demographic rates (growth, survival, and recruitment rates) are high at the most dominant elevation for each species, irrespective of the degree of local crowding. ADGR was greater for larger individuals of the two species (Figure 2a, b, Appendix S4: Figure S4.1). ADGR of the two species was decreased by the increase in local crowding (Figure 2a, b). ADGR of *A*. *veitchii*, dominating at low elevations, was greater at higher elevations (Figure 2a). On the other hand, ADGR of *A*. *mariesii*, dominating at high elevations, was lower at higher elevations, except for small individuals <10 cm DBH (Figure 2b). Therefore, the highest ADGR of the two species did not match their dominant elevations.

The mortality of the two species had a U‐shaped pattern with a peak at small and large DBH (Figure 2c, d). The mortality of the two species was increased by the increase in local crowding (Figure 2c, d). Mortality rates at higher elevations were lower and higher for *A*. *veitchii* and *A*. *mariesii*, respectively (Figure 2c, d). Therefore, the lowest mortality of the two species did not match their most dominant elevations (Figure 2c, d).

**FIGURE 3:**
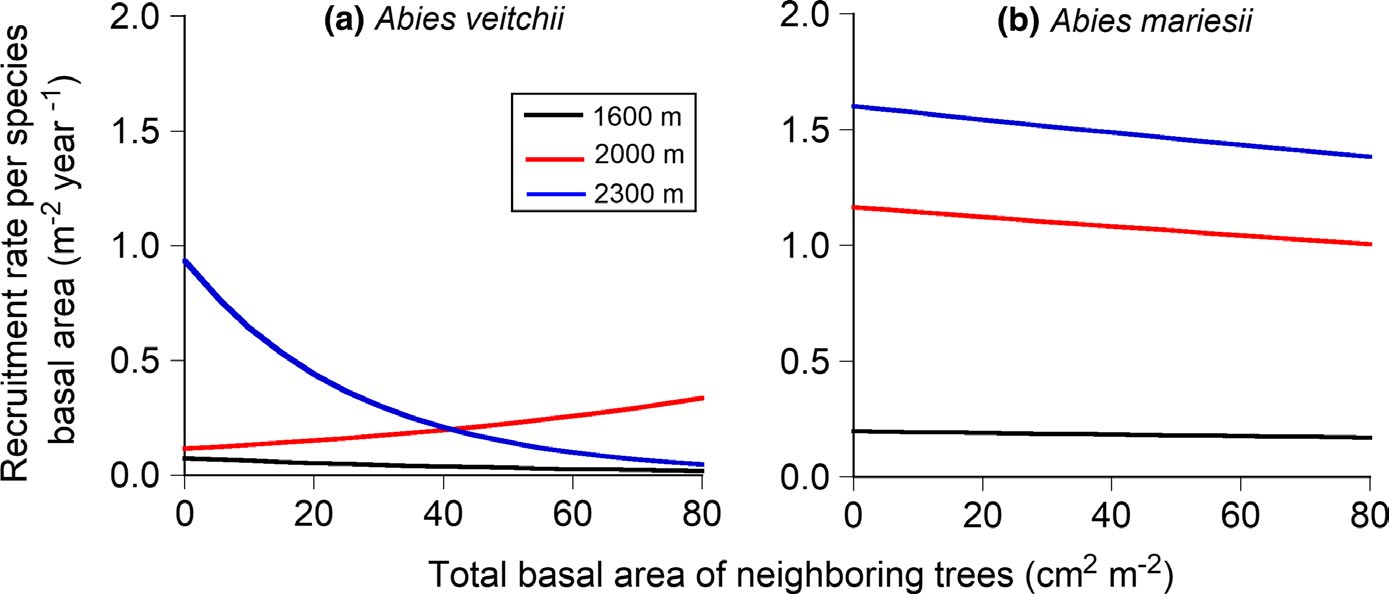
Estimated recruitment rates per species basal area to the total basal area of neighboring trees for *Abies veitchii* (a) and *A*. *mariesii* (b). Black, red, and blue lines indicate 1600 m, 2000 m, and 2300 m a.s.l., respectively. See Appendix S4: Table S4.5 for equation parameters

The recruitment rate per species basal area at zero of local crowding was greater at higher elevations for the two species (Figure 3). The recruitment rate per species basal area decreased with the increase in local crowding for the two species at three elevations, except for *A*. *veitchii* at 2000 m a.s.l. (Figure 3). The decrease of recruitment rate per species basal area due to the increase in local crowding at 2300 m a.s.l. was greater in *A*. *veitchii* than *A*. *mariesii* (Figure 3), indicating the species difference in the sensitivity of recruitment to local crowding. Recruitment rate per species basal area of *A*. *mariesii* was greater at higher elevations with any local crowding. Higher recruitment rates per species basal area at higher elevations matched the dominant elevation for *A*. *mariesii*. However, the recruitment rate of *A*. *veitchii* with any local crowding was lowest at 1600 m a.s.l., so the recruitment rate of *A*. *veitchii* did not match the most dominant elevation.

### 4.2 Growth, shade tolerance, and disturbance

While the total basal area of *A*. *veitchii* increased at 1600 m and 2000 m a.s.l. during the examined period, the tree density decreased (Appendix S4: Table S4.4). The reduction of density of small individuals was conspicuous for *A*. *veitchii* at 1600 m and 2000 m a.s.l. (Figure 1). In contrast, total basal area of *A*. *mariesii* increased at the three elevations, and the density was almost unchanged (Figure 1, Appendix S4: Table S4.4).

**FIGURE 4:**
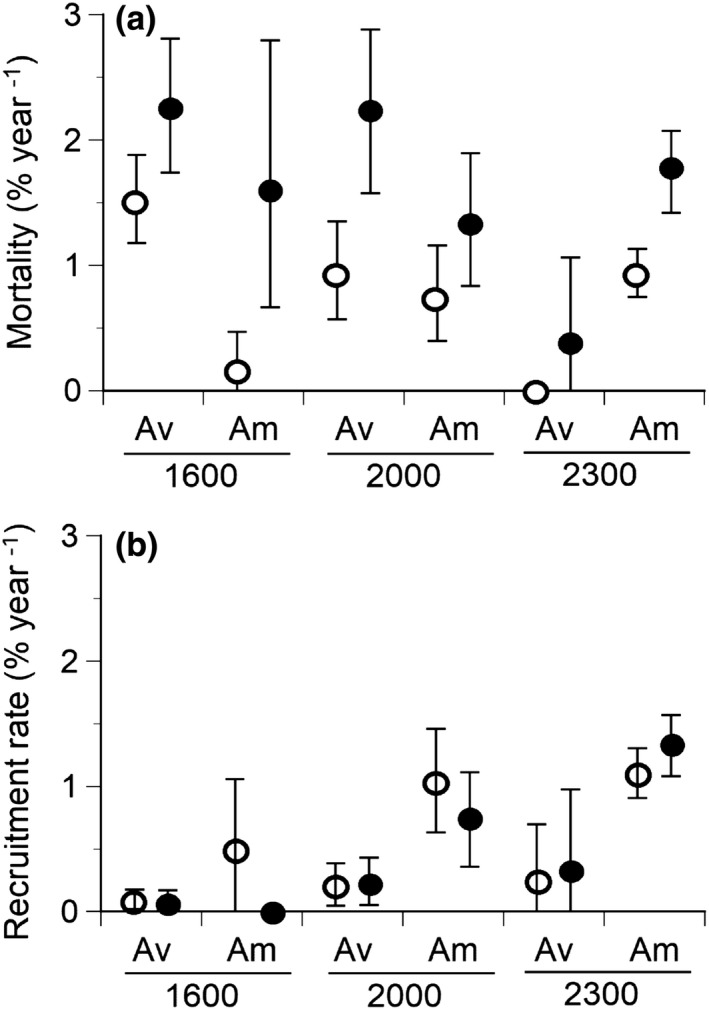
Mortalities (a) and recruitment rates (b) of *Abies veitchii* (Av) and *A*. *mariesii* (Am) at three elevations (1600 m, 2000 m, and 2300 m a.s.l.). Mean values with the 95% confidence intervals are shown. The 95% confidence interval was determined by bootstrapping. Open and solid circles indicate early period (2004‒2011 [2006‒2011 at 2000 m a.s.l.]) and latter period (2011‒2016), respectively, for each species at each elevation

The mortality (Equation 5) of the two species increased in the latter period, compared to the early period (Figure 4a). The mortality of *A*. *veitchii* was significantly greater than *A*. *mariesii* in the early period at 1600 m a.s.l. because the 95% confidence interval did not overlap between the two species (Figure 4a). On the other hand, the mortality of *A*. *mariesii* was greater than *A*. *veitchii* in both the early and latter periods at 2300 m a.s.l. (Figure 4a). Furthermore, the percentage of stem broken and uprooting (i.e., death by disturbances) to the total number of dead trees in both the early period (66%) and latter period (62%) at 2300 m was greater than that at 1600 m and 2000 m a.s.l. (6~12% in the early period, chi‐square test, $x_{2}^{2}$ = 91.3, *p* < .001, and 19~31% in the latter period, the latter period, chi‐square test, $x_{2}^{2}$ = 47.6, *p* < .001, Appendix S4: Figure S4.2).

The recruitment rate was greater for *A*. *mariesii* than *A*. *veitchii* at the three elevations in both the early and latter periods, except for no recruitment of *A*. *mariesii* in the latter period at 1600 m a.s.l. (Figure 4b). The recruitment rate tended to be greater at higher elevations for the two species (Figure 4b). Furthermore, the recruitment rate was lower than the mortality at 1600 m and 2000 m a.s.l.

**FIGURE 5:**
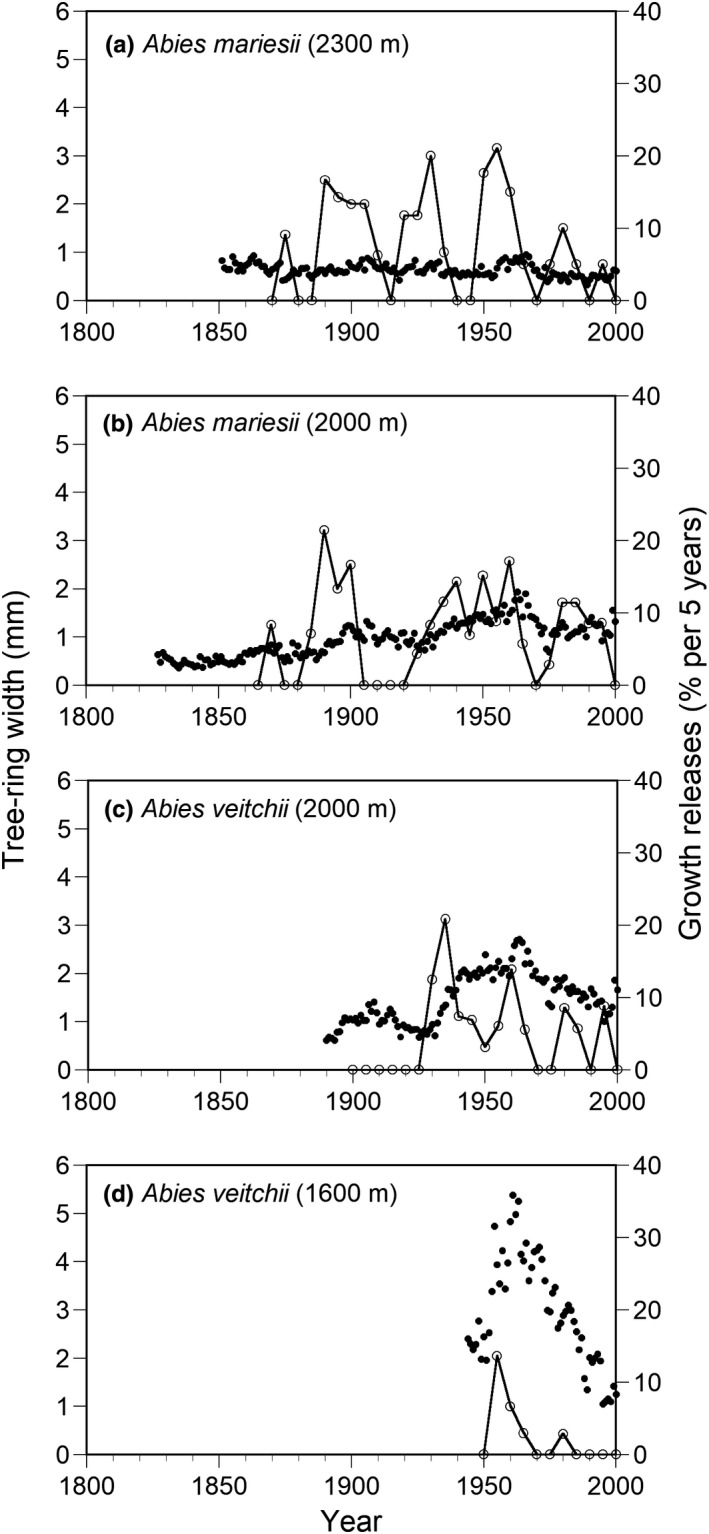
Chronologies of mean tree‐ring widths (solid circle) and percentages of growth releases (open circle) of *A*. *mariesii* at 2300 m a.s.l. (a) and 2000 m a.s.l. (b) and *Abies veitchii* at 2000 m a.s.l. (c) and 1600 m a.s.l. (d), and a tree‐ring width chronology was obtained by averaging the individual series in each year for each species at each elevation. The minimum number of trees used for each year is five

The tree‐ring width chronology could be constructed for only about 60 years, from the 1940s at 1600 m a.s.l.; nevertheless, wood cores were taken from large canopy trees (Figure 5). In contrast, the tree‐ring width chronology could be constructed for about 150 years from the 1850s at 2300 m a.s.l. Average tree‐ring width was greater at the low elevation than at the high elevation for each *A*. *veitchii* (1600 m and 2000 m a.s.l.) and *A*. *mariesii* (2000 m and 2300 m a.s.l.) (Figure 5). Synchronous growth releases in tree‐ring widths were found among individuals (Figure 5). The percentage of individuals with growth release in the 1950s among the three elevations exceeded 10% per 5 years. Therefore, large disturbances had occurred irrespective of elevations (Figure 5).

The period with growth release greater than 10% per 5 years was found at about 30 year‐intervals at 2300 m a.s.l. (1890–1910, 1920–1935, 1950–1965, 1980) (Figure 5a). However, the degree of the increase of tree‐ring width after the growth release was only a little at 2300 m a.s.l. In contrast, the degree of the increase of tree‐ring width after the growth release was more conspicuous at lower elevations. A rapid growth was observed after the growth release in the 1950s at 1600 m a.s.l., but then the growth decreased rapidly (Figure 5d). The tree‐ring width increased after the growth release in the 1950s also at 2000 m a.s.l., but then the tree‐ring width gradually decreased, and this decrease was more notable in *A*. *veitchii* than in *A*. *mariesii* (Figure 5b, c).

**FIGURE 6:**
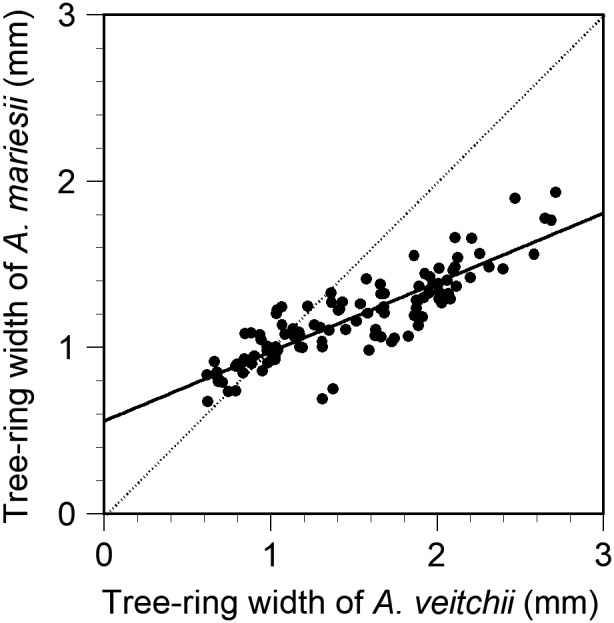
Relationship between mean tree‐ring width of *Abies veitchii* and that of *A*. *mariesii* in each year at 2000 m a.s.l. The regression equation is *Y* = 0.416 *X* + 0.557 (*R* ^2^ = 0.755, *F*_1,109_ = 335.1, *p* < .001). The 95% confidence intervals of regression slope and intercept are 0.363‒0.469 and 0.487‒0.627, respectively, which do not include 1 and 0, respectively. A dotted line indicates the 1:1 relationship (i.e., slope 1 and intercept 0)

The average tree‐ring width of *A*. *mariesii* in each year positively correlated with that of *A*. *veitchii* at 2000 m a.s.l. (Figure 6). The 95% confidence interval of the regression slope ranged between 0.363–0.469 and did not include 1.0 (Figure 6), which indicates that the growth of *A*. *mariesii* after growth releases was lower than that of *A*. *veitchii*. By contrast, the tree‐ring width of *A*. *mariesii* tended to be greater than that of *A*. *veitchii* in the range of <1 mm of tree‐ring width (Figure 6).

## 5 DISCUSSION

### 5.1 Effects of elevation and competition on three demographic rates

The recruitment rate per species basal area of *A*. *mariesii* was highest at the most dominant high elevation, while that of *A*. *veitchii* was largely decreased by local crowding (i.e., competition) at the high elevation so that the density of *A*. *veitchii* was lowest. On the contrary, the growth and survival of the two species were either not highest at the most dominant elevation and were not largely reduced by local crowding at the non‐dominant elevation. Therefore, only the recruitment rates of the two species reflected to the dominance of *A*. *mariesii* at the high elevation.

The growth rate was lower and mortality was higher at higher elevations, irrespective of the degree of local crowding, for *A*. *mariesii* greater than 10 cm DBH; nevertheless, *A*. *mariesii* dominated at high elevations (Figure 2). Many studies showed that plant growth is lower at high elevations (Coomes & Allen, 2007; Kajimoto, 1993; Mäkinen et al., 2002; Oleksyn et al., 1998; Paulsen et al., 2000; Takahashi, 2003), because the growth period is shorter at higher elevations. The growth reduction often increases mortality because of the shortage of whole‐plant carbon gain (Takahashi, 2010a). Furthermore, the growth reduction of *A*. *mariesii* at high elevations was more conspicuous for larger individuals, probably because the maintenance cost, such as respiration, is greater for larger individuals (Givnish, 1988). Therefore, the decrease of growth rates and the increase in mortality of *A*. *mariesii* at high elevations may have been caused by the decrease of whole‐plant carbon gain due to the short growth period.

In contrast, the growth was lower and the mortality was higher at lower elevations, irrespective of the degree of local crowding, for *A*. *veitchii*, which are the opposite patterns to those of *A*. *mariesii*. It is thought that lower growth rate and higher mortality of *A*. *veitchii* relate to more intense competition between trees at lower elevations because the stand basal area was greater at two lower elevations (1600 m and 2000 m a.s.l.) than at 2300 m a.s.l. due to a longer growth period and fewer occurrences of small disturbances (i.e., the mortality due to disturbances was smaller at lower elevations, Figure S4.2). *A*. *veitchii* was distributed mainly at lower elevations, so the growth and mortality of *A*. *veitchii* at low elevations largely reflected the statistical results of the three elevations (i.e., coefficients of growth and mortality equations were determined to explain the variations of trees at lower elevations). Therefore, the high growth rate and low mortality of *A*. *veitchii* at higher elevations do not mean that *A*. *veitchii* acclimated to higher elevations.

A species difference was found in response to local crowding in the recruitment rate per species basal area at the high elevation (2300 m a.s.l.). Although the recruitment rate per species basal area of *A*. *mariesii* slightly decreased with the increase in local crowding, that of *A*. *veitchii* largely decreased. The double stresses of high elevation and local crowding probably caused the large reduction of the recruitment rate of *A*. *veitchii*. Actually, the photosynthetic rate of *A*. *veitchii* decreases more at high elevations and at dark conditions than that of *A*. *mariesii* (Suzuki & Takahashi, 2020). In addition, less shade‐tolerant *A*. *veitchii* needs more carbon gain by photosynthesis to maintain the whole‐plant leaf mass, compared with shade‐tolerant *A*. *mariesii*, because of the shorter leaf longevity (Takahashi & Obata, 2014). This results in the physiological and competitive disadvantage for *A*. *veitchii* in resource‐limited environments such as dark forest understory conditions at high elevations with short growth periods (Suzuki & Takahashi, 2020). On the contrary, shade‐tolerant *A*. *mariesii* can grow and survive even in forest understory conditions at high elevations. Therefore, it may be possible that *A*. *mariesii* physiologically acclimates more to high elevations than *A*. *veitchii* and is competitively superior to *A*. *veitchii* in high elevations.

### 5.2 Growth, shade tolerance, and disturbance

The growth of trees was released around 1890–1910, 1920–1935, 1950–1965, and 1980 at the three elevations, although we have only tree‐ring data from 1940s at 1600 m a.s.l. Growth release and gap formation due to typhoons were observed at the same periods in other forests in central Japan (Kanzaki, 1984; Naka, 1982; Takahashi et al., 2018). Furthermore, many trees of the two *Abies* species died from small disturbances at high elevations during the observation period of this study, probably because of strong winds and much snow (Miyajima & Takahashi, 2007; Takahashi et al., 2012). Therefore, it is suggested that frequent small disturbances at high elevations and infrequent large disturbances due to typhoon are important factors affecting forest dynamics of the study sites.

The tree age of the most dominant *A*. *veitchii* canopy tree at 1600 m a.s.l. was about 60 years old at 1.3 m height, with only one growth release, which indicates that the disturbance about 60 years ago was so great that the whole stand was destroyed. The unimodal size structure with wide variation of *A*. *veitchii* at the low elevation (1600 m a.s.l.) indicates that many individuals were recruited during a short period after the last large disturbance, and then the recruitment gradually decreased and the forest stand developed (Takahashi, 2010b; Wright et al., 2003). Greater mortality in the latter period than the early period also supports the stand development with time. Mortality tended to be greater than the recruitment rate at lower elevations, indicating stand development after large disturbances at lower elevations. In other words, the size structure at 1600 m a.s.l. is not stable, and the stand developed, accompanied by the reduction of small individuals of *A*. *veitchii* after the last large disturbance. Although the size structure of *A*. *veitchii* at 2000 m a.s.l. had an inverse‐J‐shaped pattern, many small individuals of *A*. *veitchii* died of suppression during the measurement period (Appendix S4: Figure S4.2). In contrast, the number of small individuals of *A*. *mariesii* did not decrease at 2000 m a.s.l. as at 1600 m a.s.l. Shade‐tolerant *A*. *mariesii* can survive in dark understory conditions with less amount of carbon gain than less shade‐tolerant *A*. *veitchii* (Takahashi & Obata, 2014). Thus, *A*. *veitchii* dominated earlier than *A*. *mariesii* as a result of high growth and recruitment rates after the last large disturbance, but shade‐tolerant *A*. *mariesii* gradually increased with the progress of stand development. The long growth period at low elevations increases the interspecific difference of the annual growth rate between fast‐growing *A*. *veitchii* and slow‐growing *A*. *mariesii*. As far as the three elevations studied, it is suggested that the species composition and size structure of *Abies* forest stands are greatly affected by large disturbances at low elevations and that large disturbances are indispensable for the dominance of less shade‐tolerant *A*. *veitchii* at low elevations.

On the contrary, the inverse‐J‐shaped size structure at 2300 m a.s.l. was unchanged during the examined period, indicating a time‐stable size structure due to constant recruitment, growth rates, and mortality. The amount of carbon gain of plants decreases at high elevations because of short growth periods. However, shade‐tolerant *A*. *mariesii* can survive at high elevations more than less shade‐tolerant *A*. *veitchii* because *A*. *mariesii* can survive with less amount of carbon gain than *A*. *veitchii* (Takahashi & Obata, 2014). If slow growth of trees and frequent occurrence of small disturbances from strong winds and heavy snow prevent the development of a canopy layer at 2300 m a.s.l., then *A*. *mariesii* dominates through its high recruitment. Therefore, it is suggested that small disturbances frequently occur in higher elevations, which in turn influences the elevational distributions of the two *Abies* species through population dynamics.

A limitation of our study was that there was no replication of study plots within each elevation. The dominance of *A*. *veitchii* at lower elevations than *A*. *mariesii* is often observed in many mountains (Aizawa & Kaji, 2003). Although we found that the cause of the dominance of *A*. *veitchii* at low elevations was due to the high growth rate after a large disturbance, it is possible that shade‐tolerant *A*. *mariesii* will gradually dominate if a large disturbance does not occur. However, giving that the tree longevity of *A*. *veitchii* is longer than 200 years (Kanzaki, 1984), the interval (about 30 years) of the occurrence of large disturbances was not long in this forest stand. Therefore, *A*. *veitchii* will not be excluded by *A*. *mariesii* at low elevations. However, further studies are necessary to examine the population dynamics of the two species at many sites along elevational gradients in order to clarify whether the findings of this study are general patterns (i.e., whether *A*. *veitchii* dominates low elevations by its high growth rate after large disturbances and whether *A*. *mariesii* dominates high elevations with frequent occurrence of small disturbances by its high recruitment rate).

### 5.3 Conclusion

This study revealed the elevational distributions of the two *Abies* species from viewpoints of the population dynamics, tree competition, and disturbances using long‐term observation data (13 years) of the three permanent plots. This study showed (1) that growth and survival rates were not highest at the most dominant elevation for each species, and at the high elevation where *A*. *mariesii* dominated and small disturbances frequently occurred, the recruitment rate of *A*. *mariesii* was highest among the three elevations and that of *A*. *veitchii* was largely decreased by local crowding (i.e., competition), and (2) that *A*. *veitchii* dominated earlier than *A*. *mariesii* at low elevations because of the high growth rate after large disturbances. Therefore, *A*. *mariesii* was superior to *A*. *veitchii* at the high elevation because of its high recruitment rate and large reduction of recruitment of *A*. *veitchii* due to competition, while *A*. *veitchii* was superior to *A*. *mariesii* at the low elevation after large disturbances because of higher growth rate than *A*. *mariesii*. It is suggested that the disturbance regime changes with elevation, which further influences the elevational distributions of the two species through population dynamics in relation to competition and disturbance.

It is necessary to examine population dynamics of tree species along an elevational gradient to understand elevational vegetation distributions. Transplant experiments are often performed to examine whether species can grow and survive beyond the current upper and lower distribution limits (Angert & Schemske, 2005; Gonzalo‐Turpin & Harzald, 2009; Scheepens & Stocklin, 2013). This method provides useful information to determine whether the species can physiologically distribute outside the current distribution range. However, it is only possible to perform such experiments with seedlings and saplings. Longevity of many canopy tree species exceeds 100 years, and the maximum tree height also often exceeds 20 m. Therefore, the transplant experiments in terms of seed germination and seedling growth and survival can examine only the early stage of the life history for a tree species. Tree competition affects growth, survival, and recruitment rates from small seedlings to large canopy trees in actual forests. Furthermore, growth, survival, and recruitment rates of each species change with tree size and elevation because of ontogenic and physiological changes (Coomes & Allen, 2007; Takahashi, 2010a). Therefore, the method used in this study, based on the long‐term plot census along an elevational gradient, is important to clarify mechanisms of the elevational distribution of tree species.

## CONFLICT OF INTEREST

The authors declare no conflict of interest.

## AUTHOR CONTRIBUTIONS

**Koichi Takahashi:** Conceptualization (lead); Data curation (lead); Formal analysis (lead); Funding acquisition (lead); Investigation (lead); Methodology (lead); Project administration (lead); Resources (lead); Supervision (lead); Writing – original draft (lead); Writing – review & editing (lead). **Keigo Ikeda:** Data curation (equal); Investigation (equal). **Isao Okuhara:** Data curation (equal); Investigation (equal). **Rintaro Kurasawa:** Data curation (equal); Investigation (equal). **Suguru Kobayashi:** Data curation (equal); Investigation (equal).

## Supporting information

Appendix S1

Appendix S2

Appendix S3

Appendix S4

## Data Availability

The data used in this study is deposited in the Dryad Digital Repository: https://doi.org/10.5061/dryad.c59zw3r96.
