# Supplementary material for: Competition and disturbance affect elevational distribution of two congeneric conifers
Source: Ecol Evol. 2022 Feb 19;12(2):e8647. doi: 10.1002/ece3.8647 (PMC8858215; doi:10.1002/ece3.8647)
Supplement: Supplementary file 1 — Appendix S1 [file ECE3-12-e8647-s003.pdf]

Appendix S1

Calculation of local crowding

In this study, local crowding of neighboring trees was calculated as the sum of basal area of trees within the 10 × 10 m grid where the target tree is located. Many other studies often defined neighborhood area as a 10 × 10 m quadrat in which the target tree is located (Kohyama, 1992, 1993; Nakashizuka & Kohyama, 1995; Takahashi & Kohyama, 1999; Takahashi et al., 2018). However, this definition of neighborhood area sometimes brings about a crude measure of local crowding if target trees are near the edge of a quadrat, i.e., a target tree near the edge of a quadrat will be affected by trees at the adjacent quadrat but not much by trees at the other end of the same quadrat. Therefore, many studies also defined the neighborhood area as a certain distance from each target tree (Weiner, 1984; Takahashi, 1996; Fortunel et al., 2018). The degree of competition ability of neighboring trees for light and soil resources to the target tree decreases with increasing the distance from the target tree. Therefore, the competition ability of each neighboring tree to the target tree can be expressed by the basal area divided by the distance from the target tree (Weiner, 1984; Takahashi, 1996; Fortunel et al., 2018). The calculation method of local crowding with the distance of neighboring trees from the target tree may be more precise than that without the distance (e.g., 10 × 10 m grid).

In the preliminary analysis, this study compared the fitness of multiple regression models for the absolute diameter growth rate (ADGR) between regression models with and without the distance from target trees. Five species including *Abies mariesii* and *A. veitchii* at the three elevations (1600 m, 2000 m, and 2300 m a.s.l.) were used for the analysis. ADGR was calculated for the two periods (2004–2011 [or 2006–2011 for 2000 m a.s.l.] and 2011–2016). Tree size (DBH), elevation, and local crowding were included as independent variables of the models. Elevation was treated as a categorical variable. Local crowding was expressed as the sum of basal area of neighboring trees within the 10 × 10 m grid where the target tree was located for the model without the distance (model 1), and as the sum of basal area divided by the distance for the model with the distance (model 2). Neighboring trees were defined as any trees within 5 m from the target tree in the model 2. The multiple regression analysis was conducted for the five species at the two periods, and the results were compared between the two models. However, percentages of variations explained by the independent variables (R^2) were almost similar values between the two models for the

five species (Table S1.1). The mean R^2 values of the five species at the two periods were 0.255 and 0.257 for the model 1 and model 2, respectively. Therefore, at least, it is not shown that the model with the distance is more precise than that without the distance.

Table S1.1. Results of multiple linear regression models for two models of absolute diameter growth rates (ADGR) of five species at the three elevations (1600 m, 2000 m, and 2300 m above sea level) during two periods on Mount Norikura in central Japan. The model 2 assumes that competitive ability of neiboring trees decreases with the distance from the target tree, while the model 1 assumes that neighboring trees affect the growth of target tree, irrespective of the distance from the target tree.

**Model 1**								
		Variables					Whole model	
Species†	Period‡	Intercept	ln DBH	2000	2300	Local crowding	R^2	n
Av	1	-0.11894 ***	0.10606 ***	0.007533	0.059112 ***	-0.0006915 ***	0.409 ***	993
Av	2	-0.23167 ***	0.129198 ***	0.018027 *	0.092719 ***	-0.000222	0.391 ***	993
Am	1	0.064491 ***	0.055556 ***	-0.06897 ***	-0.06253 ***	-0.0006609 ***	0.239 ***	1570
Am	2	0.040604 **	0.054355 ***	-0.05349 ***	-0.02987 ***	-0.0008591 ***	0.209 ***	1570
Td	1	0.017226	0.044526 ***	0.006018		-0.0003774 *	0.156 ***	376
Td	2	-0.07122 ***	0.06017 ***	0.01903 **		-0.000057	0.261 ***	376
Pj	1	0.061088	0.068976 ***	-0.10301 ***	-0.07194 **	-0.000594	0.338 ***	172
Pj	2	0.023988	0.072204 ***	-0.11093 ***	-0.10466 ***	-0.000315	0.273 ***	172
Be	1	0.001317	0.079936 ***	-0.04332 *	0.002474	-0.000729	0.158 ***	363
Be	2	-0.0569	0.100937 ***	-0.03908	-0.052 *	-0.00084 *	0.12 ***	363
**Model 2**								
Variables		Variables					Whole model	
Species†	Period‡	Intercept	ln DBH	2000	2300	Local crowding	R^2	n
Av	1	-0.1231 ***	0.1068 ***	0.01365 *	0.06857 ***	-0.000024 ***	0.411 ***	802
Av	2	-0.2132 ***	0.1277 ***	0.02354 **	0.08543 ***	-0.00002 ***	0.394 ***	802
Am	1	0.07841 ***	0.05328 ***	-0.07299 ***	-0.06268 ***	-0.000026 ***	0.257 ***	1280
Am	2	0.0494 **	0.05122 ***	-0.05306 ***	-0.02952 **	-0.00003 ***	0.221 ***	1280
Td	1	0.03091	0.03747 ***	0.01062		-0.000011	0.101 ***	294
Td	2	-0.08774 ***	0.06686 ***	0.02796 ***		-0.000004	0.315 ***	294
Pj	1	0.08136	0.05917 ***	-0.1181 ***	-0.08417 **	-0.000008	0.298 ***	139
Pj	2	0.04656	0.06451 ***	-0.1478 ***	-0.127 ***	0.000004	0.251 ***	139
Be	1	-0.00351	0.08776 ***	-0.03385	-0.00033	-0.0000488 ***	0.177 ***	292
Be	2	-0.06745	0.1029 ***	-0.01282	-0.03038	0.00005 ***	0.142 ***	292

Model 1: $\mathrm{ADGR} = a_0 + a_{1j} + a_2 \ln \mathrm{DBH} + a_3 \sum \mathrm{BA}$, Model 2: $\mathrm{ADGR} = a_0 + a_{1j} + a_2 \ln \mathrm{DBH} + a_3 \sum \mathrm{BA}/d$

where $a_0 \sim a_3$ are coefficients, and ln is the natural logarithm. Coefficient a_{1j} is a categorical variable of elevation j, and coefficient a_1 of 1600 m a.s.l. is zero. Local crowding was calculated as the total basal area (cm^2) of neighboring trees within a quadrat divided by the quadrat area (100 m^2) for the model 1, and as the sum of basal area (cm^2) of each neighboring tree divided by the distance (d, m) from the target tree for the model 2. Neighboring trees were defined as any trees within 5 m from each target tree for the model 2. *Tsuga diversifolia* was not distributed at 2300 m a.s.l., so the coefficient was not shown.

† Av: *Abies veitchii*, Am: *Abies mariesii*, Td: *Tsuga diversifolia*, Pj: *Picea jezoensis* var. *hondoensis*, Be: *Betula ermanii*.

‡ Period 1: 2004~2011 (or 2006~2011 for 2000 m a.s.l.), period 2: 2011~2016.

*: $P < 0.05$, **: $P < 0.01$, ***: $P < 0.001$.

In the model 2 that neighboring trees are defined as any trees within 5 m from the target tree, trees located within 5 m from the plot edge cannot be used as target trees because local crowding cannot be calculated for these trees. The number of trees that can be used for the analysis is about 20% less in the model 2 with the distance than the model 1 without the distance. Furthermore, the local crowding is also used for the analysis of mortality and recruitment rates (equations 2 and 3 in the main text). The problem of the decrease of the number of trees is more pronounced in the analysis of mortality and recruitment rates than ADGR because of the small number of dead and recruitment trees. Therefore, this study defined neighboring trees as any trees in the same 10 × 10 m grid that the target tree was located to avoid the reduction of the number of trees that can be used for analysis.

References

Fortunel, C., Lasky, J. R., Uriarte, M., Valencia, R., Wright, S. J., Garwood, N. C., & Kraft, N. J. (2018). Topography and neighborhood crowding can interact to shape species growth and distribution in a diverse Amazonian forest. *Ecology*, 99, 2272–2283

Kohyama, T. (1992). Size-structured multi-species model of rain forest trees. *Functional Ecology*, 6, 206–212.

Kohyama, T. (1993). Size-structured tree populations in gap-dynamic forest – the forest architecture hypothesis for the stable coexistence of species. *Journal of Ecology*, 81, 131–143.

Nakashizuka, T., & Kohyama, T. (1995). The significance of the asymmetric effect of crowding for coexistence in a mixed temperate forest. *Journal of Vegetation Science*, 6, 509–516.

Takahashi, K. (1996). Plastic response of crown architecture to crowding in understorey trees of two co-dominating conifers. *Annals of Botany*, 77, 159–164.

Takahashi, K., & Kohyama, T. (1999). Size-structure dynamics of two conifers in relation to understorey dwarf bamboo: a simulation study. *Journal of Vegetation Science*, 10, 833–842.

Takahashi, K., Ikeyama, Y., & Okuhara, I. (2018). Stand dynamics and competition in a mixed forest at the northern distribution limit of evergreen hardwood species. *Ecology and Evolution*, 8, 11199–11212.

Weiner, J. (1984). Neighbourhood interference amongst *Pinus rigida* individuals. *Journal of Ecology*, 72, 183–195.
