# Supplementary material for: Competition and disturbance affect elevational distribution of two congeneric conifers
Source: Ecol Evol. 2022 Feb 19;12(2):e8647. doi: 10.1002/ece3.8647 (PMC8858215; doi:10.1002/ece3.8647)
Supplement: Supplementary file 2 — Appendix S2 [file ECE3-12-e8647-s004.pdf]

Appendix S2

Effects of species' local crowding on ADGR

The absolute diameter growth rate (ADGR) of target trees was analyzed in relation to local crowding of each species (i.e., effects of species identity). Neighborhood area was defined as a 10 × 10 m quadrat in which the target tree was located to analyze tree competition (Kohyama, 1992, 1993; Takahashi & Kohyama, 1999). All trees greater than 5 cm DBH within a 10 × 10 m quadrat were treated as neighboring trees. Local crowding was calculated as the sum of the basal area of neighboring trees for each target tree. The three plots at 1600 m, 2000 m and 2300 m a.s.l. were dominated by five species, *Abies veitchii*, *A. mariesii*, *Betula ermanii*, *Picea jezoensis* var. *hondoensis* and *Tsuga diversifolia*. Total basal area of minor species other than the five species accounted for only a few percent of the plot total in each plot. Therefore, minor species were not considered in this analysis.

A generalized linear mixed model was used to analyze whether the effect of species identity of neighboring trees on the $ADGR_i$ (cm $year^{-1}$) of species *i* was different among the five species at each elevation. The model including all variables is:

$$ADGR_i = a_0 + a_1 \ln DBH + a_2 \sum BA_{Av} + a_3 \sum BA_{Am} + a_4 \sum BA_{Td} + a_5 \sum BA_{Pj} + a_6 \sum BA_{Be}$$

where $a_0 \sim a_6$ are coefficients. Ln DBH is the ln-transformed initial DBH (cm). ∑BA (cm^2 m^{-2}) is the total basal area (cm^2) of each species divided by the quadrat area (100 m^2), and the subscript of ∑BA is abbreviations of the five species name, i.e., *A. veitchii* (Av), *A. mariesii* (Am), *B. ermanii* (Be), *P. jezoensis* var. *hondoensis* (Pj) and *T. diversifolia* (Tj). The ADGR of each target tree and local crowding were calculated, and were used in the model calculation. Individual trees were treated as a random effect.

The measurement period was divided into two periods, the early period (2004–2011 [or 2006–2011 at 2000 m a.s.l.]) and latter period (2011–2016). Regression models were developed for all possible combinations of explanatory variables without interactions. Selection of independent variables was performed by the Akaike information criteria (AIC), and the model with the lowest AIC was selected as the best. The R package glmmML was used for the analysis.

Only negative effects of *A. mariesii* and *P. ezoensis* var. *hondoensis* on ADGR of *A. mariesii* at 2300 m a.s.l. were observed (Table S2.2). ADGR was determined by only DBH for the others. Unless it is a case of allelopathy, it is unlikely that a specific

species-to-species relationship will occur because the resources used by plants are almost the same (e.g., light, soil nutrients and water) (Hubbell, Ahumada, Condit, & Foster, 2001; Ahumada, Hubbell, Condit, & Foster, 2004; Álvarez-Cansino, Schnitzer, Reid, Powers, 2015; Takahashi, Ikeyama, & Okuhara, 2018). There are no reports for allelopathy for the five species examined in this study. In fact, many cases of the growth reduction of target trees by neighboring trees relate to the size and distance of neighboring trees, not to the identity of neighboring plant species (Potvin & Dutilleul, 2009, Kaitaniemi & Lintunen, 2010). This is because competition for light and soil resources (nutrients and water) increases as size of neighboring trees is larger and the distance from the target tree is closer.

Table S2.2. Results of the generalized linear mixed model, based on AIC, for absolute diameter growth rate (ADGR) of five species at three elevations. *Tsuga diversifolia* was not distribute at 2300 m a.s.l.

Elevation	Species	Equation	*n*
2300 m	Td		
	Av	ADGR = –0.0297 + 0.0869 ln DBH	574
	Am	ADGR = 0.0723 + 0.0294 ln DBH – 0.00097 Σ BA_{Am} – 0.00091 Σ BA_{Pj}	84
	Be	ADGR = 0.1682	27
	Pj	ADGR = 0.1407	20
2000 m	Td	ADGR = –0.0101 + 0.0470 ln DBH	134
	Av	ADGR = –0.1861 + 0.1181 ln DBH	367
	Am	ADGR = –0.1207 + 0.0862 ln DBH	325
	Be	ADGR = –0.3265 + 0.1762 ln DBH	203
	Pj	ADGR = –0.1618 + 0.0979 ln DBH	40
1600 m	Td	ADGR = –0.0299 + 0.0491 ln DBH	253
	Av	ADGR = –0.1848 + 0.1133 ln DBH	574
	Am	ADGR = –0.2019 + 0.1312 ln DBH	84
	Be	ADGR = –0.3905 + 0.2158 ln DBH	27
	Pj	ADGR = –0.5673 + 0.2525 ln DBH	20

n: The number of observations.

Abies veitchii (Av), *A. mariesii* (Am), *Betula ermanii* (Be), *Picea jezoensis* var. *hondoensis* (Pj) and *Tsuga diversifolia* (Tj).

References

Ahumada, J. A., Hubbell, S. P., Condit, R., & Foster, R. B. (2004). Long-term tree survival in a neotropical forest: the influence of local biotic neighborhood. In E. Losos, E. G. Leigh Jr, & R. Condit (Eds.), *Forest diversity and dynamism: Findings from a network of large-scale tropical forest plots* (pp. 408–432). Chicago, IL: University of Chicago Press.

Álvarez-Cansino, L., Schnitzer, S. A., Reid, J. P., & Powers, J. S. (2015). Liana competition with tropical trees varies seasonally but not with tree species identity. *Ecology*, 96, 39–45.

Hubbell, S. P., Ahumada, J. A., Condit, R., & Foster, R. B. (2001). Local neighborhood effects on long-term survival of individual trees in a neotropical forest. *Ecological Research*, 16, S45–S61.

Kaitaniemi, P., & Lintunen, A. (2010). Neighbor identity and competition influence tree growth in Scots pine, Siberian larch, and silver birch. *Annals of Forest Science*, 67, 604–604.

Kohyama, T. (1992). Size-structured multi-species model of rain forest trees. *Functional Ecology*, 6, 206–212.

Kohyama, T. (1993). Size-structured tree populations in gap-dynamic forest - the forest archicture hypothesis for the stable coexistence of species. *Journal of Ecology*, 81, 131–143.

Potvin, C., & Dutilleul, P. (2009). Neighborhood effects and size-asymmetric competition in a tree plantation varying in diversity. *Ecology*, 90, 321–327.

Takahashi, K., & Kohyama, T. (1999). Size-structure dynamics of two conifers in relation to understorey dwarf bamboo: a simulation study. *Journal of Vegetation Science*, 10, 833–842.

Takahashi, K., Ikeyama, Y., & Okuhara, I. (2018). Stand dynamics and competition in a mixed forest at the northern distribution limit of evergreen hardwood species. *Ecology and Evolution*, 8, 11199–11212.
