# Supplementary material for: Competition and disturbance affect elevational distribution of two congeneric conifers
Source: Ecol Evol. 2022 Feb 19;12(2):e8647. doi: 10.1002/ece3.8647 (PMC8858215; doi:10.1002/ece3.8647)
Supplement: Supplementary file 3 — Appendix S3 [file ECE3-12-e8647-s002.pdf]

**Appendix S3**

Spatial associations among five species

The spatial distribution of the five species (*Abies veitchii*, *A. mariesii*, *Tsuga diversifolia*, *Picea jezoensis* var. *hondoensis*, *Betula ermanii*) was analyzed, using the $L$ ($r$)-function, a transformation of the $K$ ($r$)-function (Ripley 1977). The $L$ ($r$)-function is based on tree-to-tree distances ($r$). A value of the $L$ ($r$)-function equal to 0 indicates a random distribution, $L$ ($r$) > 0 for a clumped distribution, and $L$ ($r$) < 0 for a regular distribution.

We analyzed the spatial association among the five species by the $L_{12}$ ($r$)-function (Lotwick & Silverman 1982), an extension of Ripley's $K$ ($r$)-function (Ripley 1977). The $L_{12}$ ($r$)-function is based on tree-to-tree distances ($r$). A value of the $L_{12}$ ($r$)-function equal to 0 indicates a mutually independent distribution, $L_{12}$ ($r$) > 0 for a positive association, and $L_{12}$ ($r$) < 0 for a negative association. Spatial association between two populations is tested conditionally against the spatial pattern of each population, classically by shifting points of a population by a random vector over a torus, while the pattern of points of the other population is unchanged (Lotwick & Silverman 1982). The Monte Carlo simulation is used to assess the significance of deviation from the complete spatial randomness assumption, generating many random spatial patterns that provide a 99% confidence envelope. Values of the $L_{12}$ ($r$)-function higher than, equal to and lower than the 99% confidence envelopes show a positive association, a mutually independent distribution and a negative association, respectively (Lingua, Cherubini, Motta, & Nola, 2008). In this study, we calculated the $L_{12}$ ($r$)-function for the 11 combinations among the five species at1600 m and 2300 m a.s.l. and for the six combinations among the four species at 2000 m a.s.l. using 1000 simulations for each.

The initial census data (2004 at 1600 m and 2300 m a.s.l. and 2006 at 2000 m a.s.l.) were used for the calculation of both $L$ ($r$) and $L_{12}$ ($r$)-functions. The two functions were calculated between 1 m and 10 m at 1 m intervals for distance $r$ by using the package ads (Pélissier & Goreaud 2015) for free statistical software R (R ver. 3.3.3) (R Core Team 2017). The clumped and regular distributions were determined if observed patterns were above and below the 99% confidence interval, respectively, at least totaling 5 m between 1 m and 10 m in distance.

*P. jezoensis* var. *hondoensis* was randomly distributed at three elevations, the most dominant species *A. veitchii*, was also randomly distributed at 1600 m a.s.l. (Table S3.3). The other dominant species showed clumped distributions at each elevation. On the other hand, the spatial correlation among dominant species at three elevations was a

random distribution in most combinations of species (Table S3.3). The only positive correlation was found between *A. mariesii* and *T. diversifolia* at 2300 m a.s.l.

Table S3.3. Results of univariate and bivariate point pattern analyses, based on $L$ and $L_{12}$ functions respectively, at three elevations. Combinations of same species and those of two different species show the results of univariate analyses and those of bivariate analyses, respectively. *Tsuga diversifolia* was not distributed at 2300 m a.s.l.

2300 m a.s.l.

| | Td | Av | Am | Be | Pj |
|---|---|---|---|---|---|
| Td | | | | | |
| Av | | C | R | R | R |
| Am | | | C | R | + |
| Be | | | | C | R |
| Pj | | | | | R |

2000 m a.s.l.

| | Td | Av | Am | Be | Pj |
|---|---|---|---|---|---|
| Td | C | R | R | R | R |
| Av | | C | R | R | R |
| Am | | | C | R | R |
| Be | | | | C | R |
| Pj | | | | | R |

1600 m a.s.l.

| | Td | Av | Am | Be | Pj |
|---|---|---|---|---|---|
| Td | C | R | R | R | R |
| Av | | R | R | R | R |
| Am | | | C | R | R |
| Be | | | | C | R |
| Pj | | | | | R |

‘C’ and ‘R’ represent the clumped and random distributions within a species, respectively, for the univariate analysis. ‘R’ and ‘+’ represent the random distribution and positive association between two species, respectively, for the bivariate analysis.

*Abies veitchii* (Av), *A. mariesii* (Am), *Betula ermanii* (Be), *Picea jezoensis* var. *hondoensis* (Pj) and *Tsuga diversifolia* (Tj).

**References**


Lingua, E., Cherubini, P., Motta, R., & Nola, P. (2008). Spatial structure along an altitudinal gradient in the Italian central Alps suggests competition and facilitation among coniferous species. *Journal of Vegetation Science*, 19, 425–436.

Lotwick, H. W., & Silverman, B. W. (1982). Methods for analyzing spatial processes of several types of points. *Journal of the Royal Statistical Society Series B (Methodological)*, 44, 406–413.

Pélissier, R., & Goreaud, F. (2015). ads package for R: A fast unbiased implementation of the K-function family for studying spatial point patterns in irregular-shaped sampling windows. *Journal of Statistical Software*, 63, 1–18.

R Core Team. (2017). *R: A language and environment for statistical computing*. Vienna, Austria: R Foundation for Statistical Computing. https://www.R-project.org/.

Ripley, B. D. (1977). Modelling spatial patterns (with discussion). *Journal of the Royal Statistical Society Series B (Methodological)*, 39, 172–212.
