# Supplementary material for: Competition and disturbance affect elevational distribution of two congeneric conifers
Source: Ecol Evol. 2022 Feb 19;12(2):e8647. doi: 10.1002/ece3.8647 (PMC8858215; doi:10.1002/ece3.8647)
Supplement: Supplementary file 4 — Appendix S4 [file ECE3-12-e8647-s001.pdf]

Appendix 4

Table S4.4. Basal area and tree density (DBH ≥ 5 cm) of five species at three elevations (1600, 2000 and 2300 m a.s.l.) at the first census (2004 or 2006) and the final census (2016) on the east slope of Mt. Norikura in central Japan.

	BA (m^2 ha^{-1})						Density (trees ha^{-1})					
	1600 m		2000 m		2300 m		1600 m		2000 m		2300 m	
Species	2004	2016	2006	2016	2004	2016	2004	2016	2006	2016	2004	2016
Abies veitchii	41.81	42.89	16.46	18.09	2.07	2.54	713	583	713	621	58	61
Abies mariesii	3.73	4.42	12.20	13.18	26.58	28.62	91	87	590	573	1366	1341
Betula ermanii	0.40	0.48	6.77	6.75	4.80	5.73	30	27	430	340	140	150
Picea jezoensis var. *hondoensis*	1.61	1.95	3.90	3.87	6.77	7.41	26	20	87	67	122	126
Tsuga diversifolia	3.23	3.58	10.73	11.16	0	0	272	262	243	238	0	0
Others	2.53	3.18	2.55	2.50	0	0.02	176	165	379	287	0	4.00
Total	53.32	56.49	52.61	55.56	40.23	44.32	1308	1144	2442	2127	1686	1682

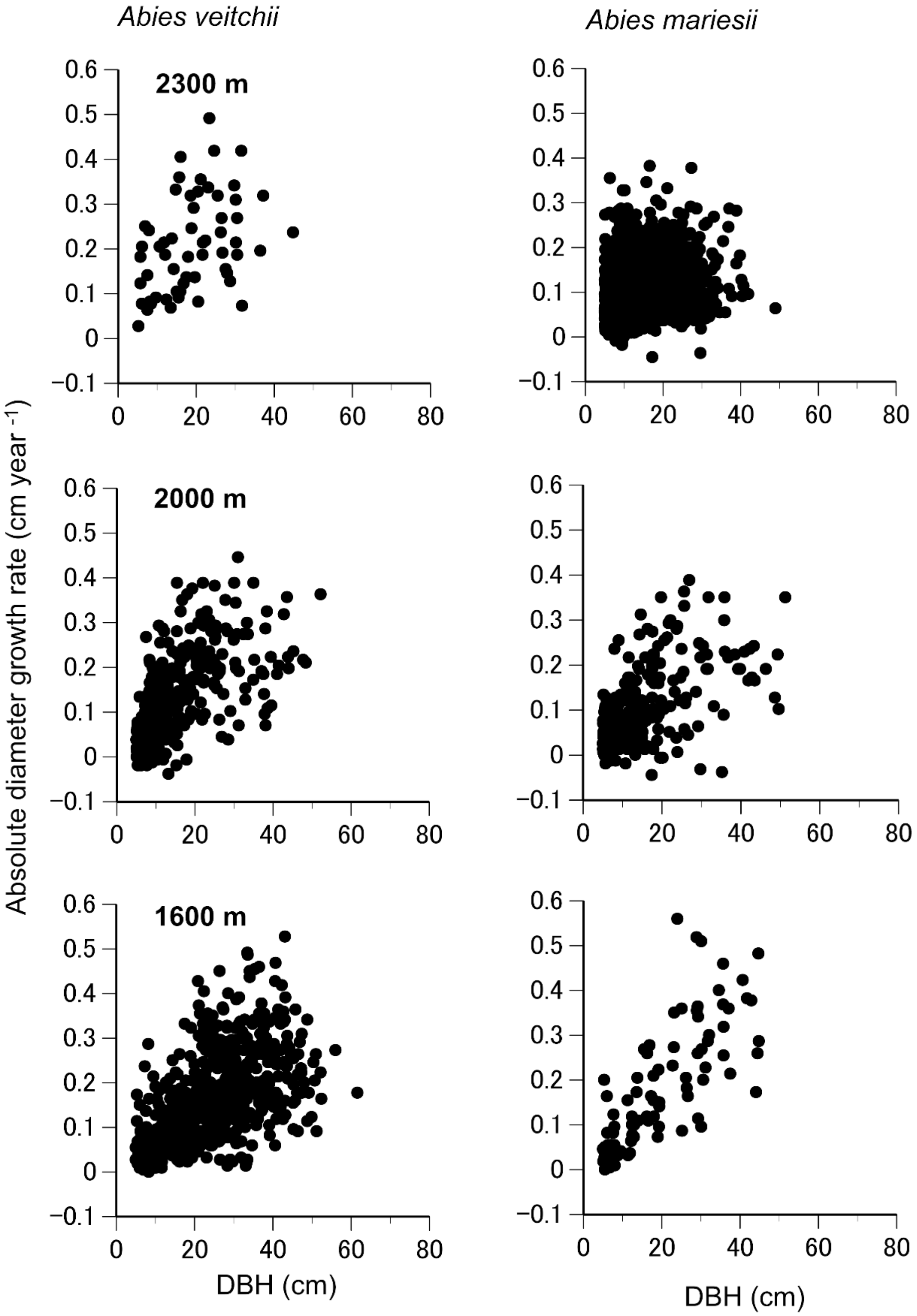

Figure S4.1. Relationships between diameter at breast height (DBH) and absolute diameter growth rate (cm year^{-1}) of *Abies veitchii* (left) and *A. mariesii* (right) during 2004–2011 at 1600 m and 2300 m a.s.l. and during 2006–2011 at 2000 m a.s.l.

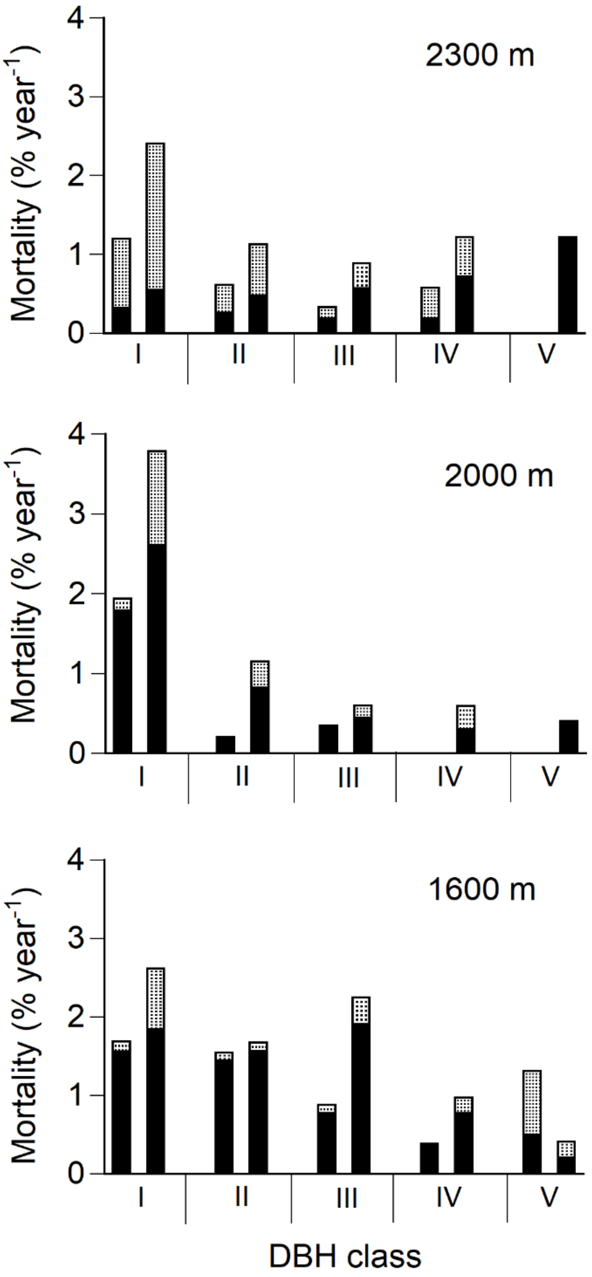

Figure S4.2. Mortality rates of all species at three elevations (1600 m, 2000 m and 2300 m a.s.l.) for five DBH classes. DBH class I: 5.0−9.9 cm, II: 10.0−19.9 cm, III: 20.0−29.9 cm, IV: 30.0−39.9 cm and V: over 40.0 cm. Solid and dotted bars indicate the mortality of standing dead trees and that of stem broken and uprooted trees, respectively. Left and right bars indicate the early period (2004–2011 [2006–2011 at 2000 m a.s.l.]) and latter period (2011–2016), respectively, for each DBH class.

Table S4.5. Results of the generalised linear mixed model for ADGR and those of the generalized linear model for mortality and recruitment rate. See equation 1, 2, and 3 in the main text. Only selected parameters, based on AIC, are shown for each species. Coefficient a_{1j}, a_{5j}, a_{6j}, b_{1j}, c_{1j}, and c_{4j} are zero for the elevation of 1600 m. Selection of parameters for ADGR, mortality and recruitment rates are shown in Table S4.6, S4.7 and S4.8, respectively.

Equation	Elevation	*A. veitchii*	*A. mariesii*
ADGR (equation 1)			
a_0		-0.1582	-0.1480
a_{1j}	2000	0.0102	0.0666
	2300	0.0740	0.2180
a_2		0.1136	0.1271
a_3		-0.0005	-0.0008
a_4			
a_{5j}	2000		-0.0415
	2300		-0.0965
a_{6j}	2000		
	2300		
n		1996	3218
Mortality (equation 2)			
b_0		-2.003052	-3.840565
b_{1j}	2000	-0.835735	0.142751
	2300	-2.799071	0.478418
b_2		-0.136247	-0.082005
b_3		0.001691	0.001304
b_4		0.005122	0.005142
b_5		0.661396	0.581838
n		2342	3689
Recruitment rate (equation 3)			
c_0		-2.200750	-1.981610
c_{1j}	2000	0.441930	1.778380
	2300	2.530410	2.096520
c_2		-0.016920	-0.001840
c_3		0.042080	0.193070
c_{4j}	2000	0.030280	
	2300	-0.020570	
n		520	520

n: The number of individual trees for ADGR and mortality, and the number of grids (10 × 10 m) for recruitment rate.

Table S4.6. Results of model selection for ADGR equation for five species. Coefficients of variables are shown. A "+" indicates the categorical variable is selected in the model. The model with the lowest AIC among the models with ΔAIC within 2 and that selected local crowding (a_3) was selected as the best model (shaded part) for *Abies veitchii* and *A. mariesii*, and was listed in Table S4.5.

Abies veitchii

#	a_0	a_{1j}	a_2	a_3	a_4	a_{5j}	a_{6j}	DF	AIC	ΔAIC
1	-0.1865	+	0.1141					6	-4238.3	0
2	-0.1582	+	0.1136	-0.00052				7	-4236.6	1.7
3	-0.1838	+	0.1132			+		8	-4223.4	15.0
4	-0.1551	+	0.1127	-0.00053		+		9	-4222.0	16.3
5	-0.1306	+	0.1045	-0.00107	0.000182			8	-4220.3	18.1

Abies mariesii

#	a_0	a_{1j}	a_2	a_3	a_4	a_{5j}	a_{6j}	DF	AIC	ΔAIC
1	-0.148	+	0.1271	-0.00079		+		9	-8560.7	0
2	-0.1551	+	0.1299	-0.00066	-5.49E-05	+		10	-8543.2	17.5
3	-0.1597	+	0.1275	-0.00059		+	+	11	-8529.3	31.4
4	-0.1677	+	0.1303	-0.00044	-5.36E-05	+	+	12	-8511.8	48.9
5	-0.1938	+		0.1286		+		8	-8503.7	57.0

Tsuga diversifolia

#	a_0	a_{1j}	a_2	a_3	a_4	a_{5j}	a_{6j}	DF	AIC	ΔAIC
1	-0.0291		0.05084					4	-2043.3	0
2	-0.02647	+	0.04765					5	-2038.2	5.1
3	-0.00866		0.04974	-0.00034				5	-2031.8	11.5
4	-0.02711	+	0.04793			+		6	-2028.9	14.4
5	-0.00719	+	0.0468	-0.00033				6	-2026.1	17.2

Picea jezoensis var. *hondoensis*

#	a_0	a_{1j}	a_2	a_3	a_4	a_{5j}	a_{6j}	DF	AIC	ΔAIC
1	-0.561	+	0.2505			+		8	-667.1	0
2	-0.5227	+	0.2475	-5.28E-04		+		9	-653.6	13.5
3	-0.5245	+	0.2482	-4.93E-04	-1.26E-05	+		10	-638.1	29.0
4	-0.5784	+	0.2518	2.44E-04	+	+		11	-627.7	39.4
5	0.02635	+	0.06804					6	-627.3	39.8

Betula ermanii

#	a_0	a_{1j}	a_2	a_3	a_4	a_{5j}	a_{6j}	DF	AIC	ΔAIC
1	-0.4392	+	0.235			+		8	-1249.4	0
2	-0.4133	+	0.2444	-8.72E-04		+		9	-1240.7	8.8
3	-0.6354	+	0.3275	2.98E-03	-1.43E-03	+		10	-1230.7	18.7
4	-0.4137	+	0.2442	-8.54E-04		+	+	11	-1212.4	37.0
5	-0.6552	+	0.338	3.25E-03	-1.58E-03	+	+	12	-1203.2	46.2

$$\mathrm{ADGR}_i = a_0 + a_{1j} + a_2 \ln \mathrm{DBH} + a_3 \sum \mathrm{BA} + a_4 \ln \mathrm{DBH} \times \sum \mathrm{BA} + a_{5j} \ln \mathrm{DBH} + a_{6j} \sum \mathrm{BA}$$

where $a_0 \sim a_{6j}$ are coefficients, ΣBA (cm^2 m^{-2}) is the total basal area (cm^2) of neighboring trees within a quadrat divided by the quadrat area (100 m^2), and ln is the natural logarithm. Coefficients a_{1j}, a_{5j}, a_{6j} are categorical variables of elevation j, and coefficient a_1 of 1600 m a.s.l. is zero.

Table S4.7. Results of model selection for mortality equation for five species. Coefficients of variables are shown. A "+" indicates the categorical variable is selected in the model. The model with the lowest AIC among the models with ΔAIC within 2 and that selected both local crowding (b_4) and observation period (b_5) was selected as the best model (shaded part) for *Abies veitchii* and *A. mariesii*, and was listed in Table S4.5.

Abies veitchii

#	b_0	b_{1j}	b_2	b_3	b_4	b_5	df	AICc	delta
1	-1.769	+	-0.1352	0.00167		0.676	6	1292.8	0
2	-2.003	+	-0.1362	0.00169	0.00512	0.661	7	1292.8	0.01
3	-0.5829	+	-0.1351	0.00169	0.00555		6	1298.5	5.7
4	-0.2955	+	-0.1339	0.00167			5	1298.9	6.03
5	-2.373	+	-0.0609			0.674	5	1300.3	7.44

Abies mariesii

#	b_0	b_{1j}	b_2	b_3	b_4	b_5	df	AICc	delta
1	-3.726		-0.0368			0.689	3	1764.8	0
2	-3.508		-0.0750	0.00108		0.701	4	1764.9	0.09
3	-3.599	+	-0.0831	0.00132		0.602	6	1765.4	0.56
4	-3.841	+	-0.0820	0.00130	0.00514	0.582	7	1765.7	0.84
5	-3.885		-0.0364		0.00350	0.688	4	1766	1.16

Tsuga diversifolia

#	b_0	b_{1j}	b_2	b_3	b_4	b_5	df	AICc	delta
1	-5.323		-0.0769			1.455	3	335.1	0
2	-4.991		-0.0769		-0.00731	1.476	4	336	0.85
3	-5.062		-0.1305	0.00149		1.484	4	336.5	1.36
4	-5.705	+	-0.0786			1.598	4	336.5	1.37
5	-5.372	+	-0.0789		-0.007513	1.623	5	337.3	2.18

Picea jezoensis var. *hondoensis*

#	b_0	b_{1j}	b_2	b_3	b_4	b_5	df	AICc	delta
1	-4.418	+	-0.2014	0.002703		2.127	6	175.1	0
2	-5.338	+	-0.0723			2.099	5	176.5	1.32
3	-4.222	+	-0.2000	0.002665	-0.00400	2.142	7	177.1	1.92
4	-5.059	+	-0.0730		-0.00540	2.119	6	178.2	3.09
5	-5.928	+		-0.00128		2.064	5	180.7	5.52

Betula ermanii

#	b_0	b_{1j}	b_2	b_3	b_4	b_5	df	AICc	delta
1	-2.317	+	-0.5180		0.02021	1.617	6	282.7	0
2	-4.560	+		-0.02831	0.02044	1.597	6	283.5	0.8
3	-2.791	+	-0.4085	-0.00599	0.02026	1.613	7	284.6	1.96
4	-1.206	+	-0.5106			1.623	5	285.4	2.77
5	-3.400	+		-0.02789		1.599	5	286.4	3.74

$$M_i = \frac{100}{1 + \exp\left(-\left(b_0 + b_{1j} + b_2 \mathrm{DBH} + b_3 \mathrm{DBH}^2 + b_4 \sum \mathrm{BA} + b_5 \ln(\mathrm{yr})\right)\right)}$$

where M_i is a percent mortality per observation period (yr, years) for species i, and $b_0 \sim b_5$ are coefficients for independent variables. Coefficient b_{1j} is a categorical variable of elevation j, and coefficient b_1 of 1600 m a.s.l. is zero. ΣBA ($cm^2\ m^{-2}$) is the total basal area (cm^2) of neighboring trees within a quadrat divided by the quadrat area ($100\ m^2$).

Table S4.8. Results of model selection for the recruitment equation of five species. Coefficients of variables are shown. A "+" indicates the categorical variable is selected in the model. The model with the lowest AIC among the models with ΔAIC within 2 and that selected both local crowding (c_2) and observation period (c_3) was selected as the best model (shaded part) for *Abies veitchii* and *A. mariesii*, and was listed in Table S4.5.

Abies veitchii

#	c_0	c_{1j}	c_2	c_3	c_{4j}	DF	AICc	ΔAIC
1	-1.941	+	-0.01703		+	6	1251.9	0
2	-2.201	+	-0.01692	0.0421	+	7	1253.7	1.9
3	-2.028	+	-0.01523			4	1273.6	21.7
4	-2.448	+	-0.01502	0.0677		5	1275.2	23.3
5	-2.813	+				3	1281.9	30.1

Abies mariesii

#	c_0	c_{1j}	c_2	c_3	c_{4j}	DF	AICc	ΔAIC
1	-2.096	+		0.1956		4	2881.5	0
2	-1.982	+	-0.00184	0.1931		5	2882.6	1.0
3	-1.515	+	-0.01107	0.1948	+	7	2882.8	1.3
4	-0.904	+				3	2907.9	26.3
5	-0.769	+	-0.00250			4	2908	26.5

Tsuga diversifolia

#	c_0	c_{1j}	c_2	c_3	c_{4j}	DF	AICc	ΔAIC
1	-7.692	+	-0.01365	1.4350		4	2850.3	0
2	-7.665	+	-0.01415	1.4350	+	5	2852.1	1.76
3	-5.018		-0.01374	1.0540		3	2883.1	32.82
4	-8.488	+		1.4500		3	2890.8	40.49
5	-5.787			1.0640		2	2924.7	74.41

Picea jezoensis var. *hondoensis*

#	c_0	c_{1j}	c_2	c_3	c_{4j}	DF	AICc	ΔAIC
1	-18.300	+	1.076E-10		+	6	1283.9	0
2	-18.790	+	0.00023	0.0795	+	7	1284.8	0.9
3	-17.760	+	-0.00856			4	1350	66.1
4	-18.330	+	-0.00812	0.1004		5	1350.2	66.3
5	-18.970	+		0.1116		4	1352.1	68.2

Betula ermanii

#	c_0	c_{1j}	c_2	c_3	c_{4j}	DF	AICc	ΔAIC
1	-18.010	+	-0.01903	0.1154		5	1369.5	0
2	-17.280	+	-0.01963			4	1370.3	0.9
3	-19.020	+	0.00034	0.1154	+	7	1373.6	4.1
4	-18.300	+	5.E-11		+	6	1374.4	4.9
5	-19.160	+		0.1412		4	1388.1	18.7

$$R_i = \sum \mathrm{BA}_i \ \exp\,(c_0 + c_{1j} + c_2 \sum \mathrm{BA} + c_3\,\mathrm{yr} + c_{4j} \sum \mathrm{BA} + 1)$$

where $c_0 \sim c_{4j}$ are coefficients of independent variables. R_i is the recruitment rate (trees ha^{-1}) of species *i* per observation period (yr, years), ΣBA (cm^2 m^{-2}) is the total basal area (cm^2) of neighboring trees within a quadrat divided by the quadrat area (100 m^2). ΣBAi (cm^2 m^{-2}) is the total basal area of species *i* in the whole plot divided by the plot area. Coefficient c_{1j} and c_{4j} are categorical variables of elevation *j*, and these coefficients are zero for 1600 m a.s.l.
